# Developmental Programming of Drug Response: Microbiota as a Missing Dimension in Perinatal Drug Discovery

**DOI:** 10.3390/ijms27114667

**Published:** 2026-05-22

**Authors:** Yanan Zhang, Liangkun Ma, Yan Wang

**Affiliations:** 1 State Key Laboratory of Bioactive Substance and Function of Natural Medicines, Institute of Materia Medica, Chinese Academy of Medical Sciences & Peking Union Medical College, Beijing 100050, China; 2 National Clinical Research Center for Women’s Health and Obstetric and Gynecologic Diseases, Department of Obstetrics and Gynecology, Peking Union Medical College Hospital, Chinese Academy of Medical Sciences & Peking Union Medical College, Beijing 100050, China

**Keywords:** perinatal pharmacology, gut microbiota, developmental programming, placenta, gestational windows, infant drug response

## Abstract

Drug exposure during pregnancy and early life is typically considered a short-term clinical intervention rather than a determinant of long-term pharmacological outcomes. Consequently, the developmental context is largely absent from drug discovery and drug development paradigms, where efficacy, safety and target engagement are evaluated predominantly in adult, steady-state systems. This disconnect may contribute to unexplained variability in drug response and toxicity later in life. Pregnancy is accompanied by dynamic remodeling of the maternal gut microbiota and its metabolic output, generating bioactive microbial metabolites that regulate immune tone, metabolic homeostasis and the expression of drug-metabolizing enzymes and transporters. These microbial signals intersect with pharmacological interventions across gestation, shaping maternal pharmacokinetics, placental regulation and fetal drug exposure during developmentally sensitive windows. Importantly, microbiota–drug interactions initiated during pregnancy do not terminate at birth. Instead, they extend into infancy through vertical microbial transmission, breast milk-mediated metabolic signaling, and the immaturity of neonatal drug-handling systems, collectively contributing to developmental programming of drug responsiveness beyond early life. In this review, we propose a microbiota-informed framework that reframes perinatal drug exposure as a developmentally embedded signal operating across a maternal–placental–infant continuum. This perspective introduces a missing developmental dimension into drug discovery and highlights new opportunities to improve translational predictability and precision pharmacotherapy across the life course.

## 1. Introduction: Drug Response as a Developmental Outcome

Pregnancy and early life constitute a uniquely sensitive yet highly plastic phase of human development, during which environmental signals exert effects that are often disproportionate in magnitude and durability relative to their duration. Pharmacological interventions administered during this period have traditionally been evaluated within a risk–benefit framework centered on maternal safety and the avoidance of acute fetal toxicity. This paradigm has successfully reduced catastrophic teratogenic outcomes, but it implicitly treats pregnancy as a temporally bounded pharmacological state and assumes that drug-related biological effects largely resolve with drug clearance or delivery [1,2,3].

However, this view has become increasingly misaligned with contemporary insights from developmental biology, systems pharmacology, and host–microbe research. A growing body of evidence indicates that drug exposure during pregnancy can induce persistent alterations in metabolic regulation, immune function, and organ development in offspring, even in the absence of overt teratogenicity. These long-term consequences are often subtle, probabilistic, and context-dependent, rendering them difficult to detect within conventional safety frameworks focused on short-term outcomes. As a result, current approaches to perinatal pharmacology may systematically underestimate the enduring biological impact of pharmacological exposure during early development [4,5,6,7].

Concurrently, the gut microbiota has emerged as a critical regulator of drug disposition, efficacy, and toxicity across the life span. Microbial enzymes directly transform xenobiotics, while microbiota-derived metabolites, including short-chain fatty acids, bile acids, and tryptophan derivatives, act as systemic signaling molecules that modulate host drug-metabolizing enzymes, transporters, immune tone, and endocrine pathways [8]. Importantly, these microbial influences are not static. During pregnancy, profound hormonal, immunological, and metabolic adaptations reshape the maternal gut microbiota and its metabolic output in a gestational stage-specific manner, thereby redefining the biological context in which pharmacological agents are absorbed, metabolized, and interpreted by host tissues [9,10].

Despite these advances, microbiota–drug interactions remain poorly integrated into perinatal pharmacology. Obstetric pharmacology, placental biology, and pediatric drug research are still largely compartmentalized, with limited consideration of biological continuity across pregnancy and early life. This compartmentalization obscures the possibility that transient pharmacological exposures during pregnancy may act as developmental signals, shaping long-term drug responsiveness and disease susceptibility in offspring through microbiota-dependent mechanisms [11,12,13].

In this review, we synthesize emerging mechanistic and clinical evidence to propose a reframing of perinatal pharmacology as a microbiota-informed, developmentally embedded process. We argue that drug exposure during pregnancy and early infancy should be understood not as a transient clinical event but as a determinant of lifelong pharmacological trajectories operating across a maternal–placental–fetal–infant continuum [14,15,16,17]. By integrating gestational timing, microbial metabolic context, and placental regulation into pharmacological thinking, this perspective challenges static exposure-based risk assessment and highlights new opportunities for precision pharmacotherapy that balances immediate maternal needs with long-term offspring health [18,19].

## 2. Methodology

This article is a narrative review that aims to integrate multidisciplinary evidence from microbiome science, perinatal pharmacology, placental biology, and developmental biology to propose an integrative conceptual framework.

### 2.1. Literature Search Strategy

A comprehensive literature search was conducted across the following databases: PubMed/MEDLINE, Web of Science Core Collection, Scopus, and Embase. Supplementary searches were performed using Google Scholar and ClinicalTrials.gov to identify recent preprints, ongoing trials, and registered clinical studies. The search covered publications from January 2000 to October 2025, with priority given to studies published within the past ten years to reflect the most current understanding of microbiota–drug interactions. The search strategy utilized combinations of the following keywords: “perinatal pharmacology”, “gut microbiota”, “developmental programming”, “placental transfer”, “microbial metabolites”, “pregnancy”, and “infant drug response”. Reference lists of key articles and relevant review papers were additionally screened to identify pertinent studies not captured by the primary database search.

### 2.2. Inclusion and Exclusion Criteria

Inclusion criteria were as follows: (i) peer-reviewed original research articles, mechanistic studies, clinical investigations, meta-analyses, and authoritative reviews published in English; (ii) studies addressing microbiota–drug interactions, placental drug handling, perinatal developmental programming, or maternal–infant microbial transmission; (iii) studies with sufficient methodological detail to permit assessment of evidence quality and translational relevance. Exclusion criteria included: (i) conference abstracts, editorials, commentaries, and non-peer-reviewed gray literature; (ii) studies unrelated to perinatal windows or microbiota–drug interactions; (iii) studies with insufficient methodological information to determine evidence level; and (iv) non-English publications without accessible English translations.

### 2.3. Study Selection and Evidence Stratification

Literature screening was performed independently by two authors (Y.Z. and L.M.), with discrepancies resolved through discussion and final adjudication by the corresponding author. Titles and abstracts were initially screened for relevance, followed by full-text evaluation of potentially eligible studies. To enhance transparency regarding the strength of supporting evidence, included studies were categorized into three tiers: (i) human evidence, comprising clinical trials, observational cohorts, and ex vivo human tissue studies; (ii) animal and in vitro evidence, including rodent models, germ-free systems, organoid studies, and cell-based mechanistic investigations; and (iii) mechanistic hypotheses, representing inferential or conceptual extensions derived from converging lines of indirect evidence.

Because the objective of this review is to construct an integrative cross-disciplinary framework, formal PRISMA systematic review procedures and quantitative risk-of-bias assessments were not applied. Nonetheless, the search strategy, selection process, and evidence stratification were designed to ensure methodological transparency and reproducibility consistent with best practices for narrative reviews in biomedical sciences.

## 3. Conceptual Framework: Microbiota-Informed Perinatal Pharmacology as a Developmental Continuum

### 3.1. Pregnancy Is Not a Transient Pharmacological State

Traditional models of perinatal pharmacology conceptualize pregnancy as a discrete physiological modifier of drug disposition, characterized by changes in plasma volume, renal clearance, and hepatic enzyme activity. Within this framework, pharmacokinetic alterations are treated as largely reversible adaptations that normalize following delivery. While such models capture important aspects of maternal physiology, they provide an incomplete representation of the biological system in which drugs act during pregnancy [20,21].

Instead, pregnancy is a systems-level developmental state marked by coordinated remodeling of endocrine signaling, immune tolerance, metabolic homeostasis, and organ function. These host-level adaptations unfold in parallel with dynamic restructuring of the maternal gut microbiota and its metabolic capacity. Far from being a passive bystander, the gut microbiota functions as a metabolically active organ that contributes to xenobiotic transformation and generates bioactive metabolites capable of regulating host transcriptional and epigenetic programs. Consequently, drug exposure during pregnancy occurs within a dynamic host–microbe ecosystem whose regulatory properties vary across gestation [22,23,24].

Crucially, the biological consequences of pharmacological exposure during pregnancy are not necessarily constrained by pharmacokinetic half-lives. Based on convergent evidence from rodent developmental models and ex vivo human placental studies, we hypothesize that drugs and their microbiota-modulated metabolites intersect with developmental processes operating within restricted windows of plasticity, during which transient perturbations may induce durable biological change. It should be noted, however, that direct longitudinal human evidence demonstrating drug–microbiota-mediated developmental programming across pregnancy and into childhood remains scarce, and the framework presented here is therefore best regarded as hypothesis-generating. From this perspective, pregnancy should not be regarded as a self-contained pharmacological episode but as a critical segment within a longer developmental trajectory in which drug exposure has the potential to become biologically embedded [25].

### 3.2. The Maternal–Placental–Fetal–Infant Axis

A defining feature of microbiota-informed perinatal pharmacology is recognition of a continuous maternal–placental–fetal–infant axis through which biological signals are transmitted across developmental stages. Within this axis, the placenta occupies a central integrative position, translating maternal-derived cues, including pharmacological agents, endogenous hormones, immune mediators, and microbiota-derived metabolites, into a regulated fetal biochemical environment [26,27,28].

Rather than serving as a passive barrier, the placenta dynamically controls fetal exposure through coordinated regulation of transporter expression, metabolic enzyme activity, receptor signaling, and epigenetic modification. Circulating microbial metabolites, such as short-chain fatty acids and bile acid derivatives, are increasingly recognized as modulators of these placental processes. Through such mechanisms, maternal gut microbial metabolism can indirectly shape fetal exposure landscapes even in the absence of direct microbial colonization of placental tissue [29].

Importantly, this axis does not terminate at birth. Vertical transmission of maternal microbiota during delivery, breast milk-mediated transfer of microbial metabolites and immune factors, and the immaturity of neonatal drug-handling systems extend prenatal biological influences into infancy. As a result, infant drug responsiveness reflects not only developmental immaturity but also prenatal microbial and pharmacological history. This continuity challenges conventional boundaries separating obstetric and pediatric pharmacology and underscores the need for integrated models that span pregnancy and early life [30].

### 3.3. Critical Windows and Developmental Programming of Drug Response

Developmental biology has long established that environmental exposures exert maximal and often irreversible effects during restricted critical windows. Increasing evidence suggests that pharmacological exposures, particularly when modulated by microbiota-derived metabolic signals, operate within these same windows to influence long-term biological trajectories. During pregnancy, such windows correspond to key processes, including placental development, organogenesis, immune system calibration, and metabolic set-point establishment [31,32].

**Figure 1:**
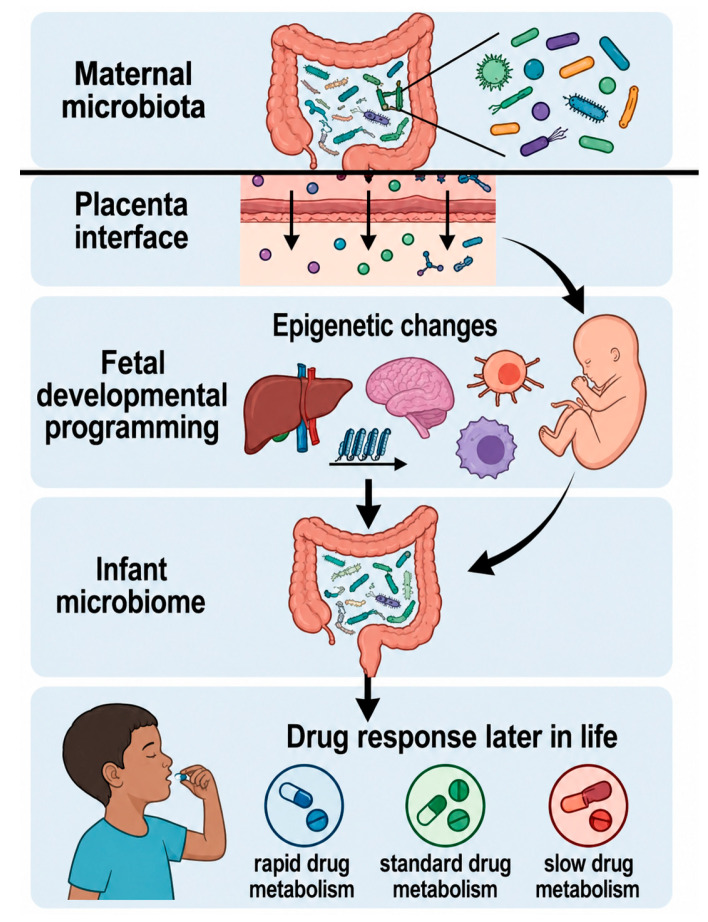
Microbiota-Informed Perinatal Pharmacology Framework (Original schematic created by the authors).

Microbiota-derived metabolites can modulate gene expression, epigenetic regulation, and cellular differentiation pathways within these windows, thereby shaping host responses to pharmacological agents. When drug exposure intersects with these microbiota-dependent regulatory processes, its biological impact may persist long after the drug has been eliminated. Notably, such programming effects may not manifest as immediate pathology but instead alter susceptibility to disease or modify drug responsiveness later in life [33,34,35,36,37]. As shown in Figure 1, the schematic illustrates the mechanistic cascade linking the maternal gut microbiota, microbial metabolites, and the placental interface to fetal development, which subsequently shapes the infant microbiota and ultimately influences drug responses later in life.

## 4. Gestational Stage-Specific Remodeling of Microbiota–Drug Interactions

Pregnancy is not a homogeneous pharmacological state but a temporally structured developmental continuum characterized by shifting physiological priorities. Across early, mid, and late gestation, coordinated changes in immune tone, metabolic capacity, and organ function redefine how pharmacological agents are absorbed, metabolized, and biologically interpreted. These host-level adaptations occur in parallel with gestational stage-specific remodeling of the maternal gut microbiota and its metabolic output, creating distinct microbial–pharmacological contexts across pregnancy.

In addition to acting through bioactive metabolites, the maternal gut microbiota directly transforms xenobiotics through a diverse enzymatic repertoire. Bacterial β-glucuronidases hydrolyze hepatically formed glucuronide conjugates, driving enterohepatic recirculation and prolonging the systemic exposure of drugs such as mycophenolate, irinotecan-derived SN-38, and certain NSAIDs. Azoreductases (AzoR), produced predominantly by *Clostridium*, *Eubacterium*, and *Enterococcus* lineages, cleave azo bonds to activate prodrugs, including sulfasalazine and balsalazide. Nitroreductases activate metronidazole and nitrazepam, while bile salt hydrolases generate unconjugated bile acids that subsequently serve as FXR/PXR ligands regulating host drug-metabolizing enzymes. Although direct longitudinal measurements of these enzymatic activities across human gestation remain scarce, converging evidence suggests trimester-dependent fluctuation. In early pregnancy, relatively preserved obligate anaerobe populations maintain baseline β-glucuronidase and BSH activity, with only modest functional remodeling. During mid-pregnancy, the shift toward carbohydrate fermentation and bile acid reconfiguration is accompanied by measurable changes in BSH activity and altered β-glucuronidase output, potentially modifying the enterohepatic recirculation of conjugated drugs and contributing to the observed variability in clearance of agents such as lamotrigine and glyburide. In late pregnancy, depletion of butyrate-producing Firmicutes and expansion of Proteobacteria may reduce overall reductive enzyme diversity while increasing exposure to LPS-associated pro-inflammatory signaling, thereby compounding host-level changes in drug disposition.

As a consequence, microbiota–drug interactions during pregnancy are inherently time-dependent. Identical pharmacological exposures may exert qualitatively different biological effects depending on when they occur, not only because of fetal developmental stage but also because of dynamic changes in microbial metabolic capacity and host responsiveness. Recognizing this temporal structure is essential for understanding variability in drug response and for identifying developmental windows during which pharmacological perturbations may become durably embedded, as shown in Table 1.

### 4.1. Early Pregnancy: Immune Tolerance, Microbial Metabolites, and Pharmacological Vulnerability

Early pregnancy encompasses implantation and early placentation, processes that require finely tuned immunological adaptation and tissue remodeling. During this phase, maternal physiology transitions from a pro-inflammatory state that facilitates implantation to an immune-tolerant environment that supports fetal survival. This recalibration is highly sensitive to metabolic and signaling cues, rendering early pregnancy a critical window for pharmacological susceptibility [38,39].

Contrary to later gestational stages, early pregnancy is not consistently associated with large-scale changes in microbial diversity. Instead, functional remodeling of microbial metabolism predominates. Subtle shifts in the production of short-chain fatty acids and tryptophan-derived metabolites occur in response to rising progesterone and estrogen levels, preceding overt taxonomic restructuring. These functional changes are biologically consequential, as microbial metabolites serve as regulators of immune tolerance, epithelial integrity, and host transcriptional programs [40].

Short-chain fatty acids promote the differentiation and expansion of regulatory T cells and suppress excessive inflammatory signaling, thereby supporting immune tolerance at the maternal–fetal interface. Simultaneously, these metabolites influence intestinal barrier function and the expression of drug transporters, potentially modifying oral drug absorption. Tryptophan-derived microbial metabolites, acting through aryl hydrocarbon receptor signaling, modulate both immune pathways and the transcriptional regulation of drug-metabolizing enzymes. During early pregnancy, when hepatic and intestinal drug-handling systems have not yet undergone full gestational adaptation, such microbiota-derived signals may exert a disproportionately large influence on pharmacokinetic variability [41].

Importantly, pharmacological exposure during early pregnancy may perturb microbial metabolic signaling even in the absence of direct embryotoxicity. Disruption of metabolite-mediated immune tolerance or epithelial regulation may alter placental establishment and early developmental trajectories, generating long-term consequences that are not captured by conventional safety endpoints. These considerations highlight early pregnancy as a window in which timing and microbial context may outweigh dose in determining biological outcomes [42,43,44].

### 4.2. Mid-Pregnancy: Microbiota Bile Acid Signaling and Metabolic Reprogramming of Drug Disposition

Mid-pregnancy is characterized by a pronounced shift toward an anabolic metabolic state designed to support rapid fetal growth and placental maturation [45]. Maternal insulin resistance increases, lipid metabolism is restructured, and energy storage pathways are upregulated. From a pharmacological perspective, this stage marks the onset of systematic remodeling of drug disposition, with emerging changes in hepatic enzyme activity, intestinal absorption, and bile acid circulation [46].

During this period, the maternal gut microbiota undergoes functional remodeling that preferentially affects bile acid metabolism and carbohydrate fermentation, even when overall taxonomic diversity remains relatively stable. Alterations in microbial bile salt hydrolase activity and secondary bile acid production reshape the circulating bile acid pool, modifying the signaling properties. Because bile acids act as ligands for nuclear receptors such as farnesoid X receptor and pregnane X receptor, microbiota-driven changes in bile acid composition directly intersect with host pathways that regulate drug-metabolizing enzymes and transporters [47].

Activation or suppression of bile acid-responsive nuclear receptors influences the transcription of cytochrome P450 enzymes, conjugation pathways, and efflux transporters in hepatic and intestinal tissues. As a result, microbiota-dependent modulation of bile acid signaling introduces an additional layer of variability into drug clearance and bioavailability during mid-pregnancy. Pharmacological agents targeting metabolic pathways, including antidiabetic and lipid-modulating drugs, are particularly sensitive to this regulatory network, as their efficacy and metabolism are closely linked to bile acid-dependent signaling pathways [48,49].

These interactions underscore the inadequacy of static dosing strategies across gestation. Drug regimens initiated or continued during mid-pregnancy may require reassessment as microbiota bile acid signaling evolves. Importantly, disruption of this axis, whether through pharmacological intervention or dietary perturbation, may exacerbate metabolic dysregulation and influence placental nutrient transport, thereby contributing to long-term metabolic programming in offspring [50,51].

### 4.3. Late Pregnancy: Microbiota Instability, Inflammation-like Physiology, and Variability in Fetal Exposure

Late pregnancy is associated with a progressive shift toward an inflammation-like physiological state that facilitates parturition and tissue remodeling. This state is characterized by increased inflammatory signaling, altered vascular dynamics, and accelerated drug clearance driven by expanded plasma volume, enhanced renal filtration, and changes in hepatic enzyme activity. These host-level adaptations present significant challenges for pharmacological management, particularly for drugs with narrow therapeutic windows [52,53].

Concurrently, late gestation is marked by increased instability of the gut microbiota. Reductions in beneficial short-chain fatty acid-producing taxa and enrichment of bacteria associated with inflammatory signaling have been reported, accompanied by altered microbial metabolite profiles. Decreased availability of anti-inflammatory metabolites such as butyrate may compromise intestinal barrier integrity and amplify systemic inflammatory tone, reinforcing the inflammation-like state of late pregnancy [54].

The convergence of microbiota instability and host inflammatory signaling has important implications for drug metabolism and placental transfer. Pro-inflammatory cytokines can modulate the expression and activity of drug-metabolizing enzymes and transporters, while altered microbial metabolite signaling may reduce regulatory buffering of hepatic and intestinal function. Together, these processes contribute to heightened interindividual variability in pharmacokinetics and fetal exposure during late gestation [55,56].

From a developmental perspective, late pregnancy remains a period of vulnerability despite proximity to delivery. Altered placental transporter expression or barrier function during this stage may influence fetal exposure to pharmacological agents in ways that are not predictable from maternal plasma concentrations alone. These observations reinforce the need to consider late pregnancy not merely as a period of accelerated clearance but as a dynamic microbial–host–placental interface with implications for developmental programming [57,58,59].

**Figure 2:**
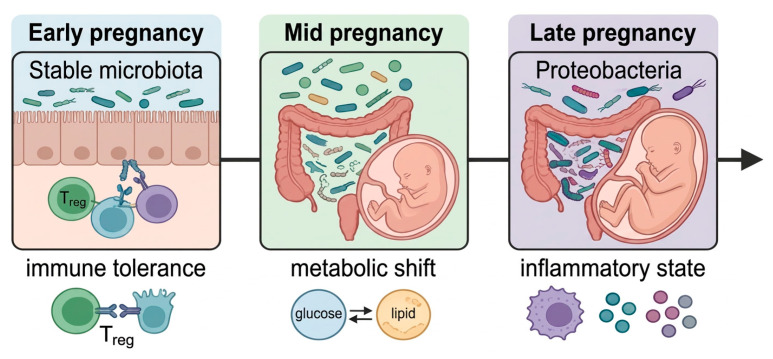
Gestational-Stage Microbiota Remodeling (Original schematic created by the authors).

As shown in Figure 2, the stage-specific remodeling of the maternal gut microbiota during pregnancy was illustrated. In early pregnancy, the maternal immune system shifts toward an immune-tolerant state to support embryo implantation and early fetal development, during which the microbial community remains relatively stable. During mid-pregnancy, metabolic adaptation becomes the dominant physiological feature, accompanied by alterations in microbial composition that promote energy harvest and metabolic flexibility. In late pregnancy, the maternal physiological state gradually transitions toward a mild pro-inflammatory condition, which is associated with further restructuring of the gut microbiota and enrichment of taxa related to inflammatory responses and energy storage. Overall, these stage-dependent microbial changes contribute to maternal metabolic adaptation and may influence fetal development through microbe-derived metabolites and maternal–fetal signaling pathways. 

## 5. The Placenta as an Integrative Interface for Microbial and Pharmacological Signals

The placenta occupies a central position within microbiota-informed perinatal pharmacology, acting as an active regulatory interface rather than a passive conduit. It integrates maternal-derived pharmacological agents, endogenous hormones, immune mediators, and microbiota-derived metabolites into a tightly regulated fetal biochemical environment. Understanding placental function is therefore essential for interpreting how maternal drug exposure translates into fetal exposure and developmental outcomes.

### 5.1. Placental Transport and Metabolism Are Dynamic and Context-Dependent

Placental drug transfer is governed by a coordinated network of transporters and metabolic enzymes, including ATP-binding cassette transporters, solute carrier proteins, and phase I and II metabolic systems. The expression and activity of these components are developmentally regulated and responsive to endocrine, metabolic, and inflammatory cues. Consequently, placental handling of drugs is not fixed but adapts to maternal physiological state [60,61].

Microbiota-derived metabolites have emerged as modulators of these placental processes. Short-chain fatty acids can influence histone acetylation and transcriptional activity in trophoblast cells, providing a potential epigenetic mechanism for regulation of transporter expression. Bile acid derivatives, acting through nuclear receptor signaling pathways, have been implicated in placental development and nutrient transport. Alterations in maternal microbial metabolism that reshape circulating metabolite profiles may therefore indirectly modify placental drug transfer capacity [62,63].

These findings challenge the assumption that fetal drug exposure can be inferred directly from maternal plasma concentrations. Instead, fetal exposure reflects an integrated outcome of maternal pharmacokinetics, placental regulation, and microbial metabolic context [64,65,66].

### 5.2. Microbiota-Driven Inflammatory Signaling and Placental Barrier Function

Beyond transport and metabolism, placental barrier integrity represents a critical determinant of fetal protection. Subclinical placental inflammation has been associated with altered permeability and dysregulated transporter expression, even in the absence of overt pathology. Maternal gut microbiota composition contributes to systemic inflammatory tone through the production of microbial metabolites and, under conditions of dysbiosis, increased exposure to pro-inflammatory microbial components [67,68].

Microbiota-associated inflammatory signaling may propagate to the placental interface, influencing trophoblast function and barrier properties. Reduced availability of anti-inflammatory metabolites, such as butyrate, may diminish protective regulatory inputs, while increased pro-inflammatory signaling may compromise placental selectivity. These effects are likely to be subtle and context-dependent, yet they may exert meaningful influence on fetal exposure during sensitive developmental windows [69,70]. Several pharmacological classes have been documented to undergo altered placental transfer under conditions of subclinical, microbiota-associated inflammation. Substrates of P-glycoprotein (ABCB1) and breast cancer resistance protein (ABCG2), including a number of antiretrovirals (e.g., darunavir, dolutegravir), glucocorticoids (dexamethasone, betamethasone), and certain antidepressants (citalopram, paroxetine), exhibit increased fetal-to-maternal transfer ratios when placental efflux transporter expression is suppressed by pro-inflammatory cytokines such as IL-6 and TNF-α, whose circulating levels are partly governed by microbial metabolite balance (notably reduced butyrate and elevated LPS exposure). Conversely, β-lactam antibiotics and certain anticonvulsants (lamotrigine, levetiracetam), which rely on solute carrier (SLC) mediated uptake, may show reduced transplacental flux when inflammation downregulates SLC transporters. Opioid analgesics (e.g., buprenorphine, methadone), partially handled by P-gp, have likewise been associated with elevated fetal exposure indices in pregnancies complicated by gut-derived endotoxemia or gestational dysbiosis. These examples concretize the mechanistic argument by demonstrating that microbiota-driven inflammatory shifts can produce clinically meaningful alterations in fetal exposure even when maternal plasma concentrations remain within target ranges [71].

Importantly, such mechanisms operate independently of the existence of a placental microbiome. Circulating microbial metabolites act as systemic mediators, linking maternal gut ecology to placental physiology without requiring direct microbial colonization [72,73].

### 5.3. Implications for Fetal Exposure and Developmental Programming

By integrating microbial, metabolic, and pharmacological signals, the placenta plays an active role in shaping fetal developmental trajectories. Microbiota-modulated alterations in placental drug handling may influence neurodevelopment, metabolic set-point establishment, and immune maturation, contributing to long-term variability in drug responsiveness and disease susceptibility [74,75].

These programming effects are unlikely to manifest as acute toxicity and may therefore evade detection within conventional obstetric monitoring. Recognizing the placenta as an active participant in microbiota–drug interactions reframes fetal pharmacological exposure as a regulated, context-dependent process rather than a passive consequence of maternal treatment. This perspective underscores the importance of incorporating placental assays and microbial metabolic profiling into perinatal pharmacology research [76].

## 6. Postnatal Extension of Microbiota–Drug Interactions to Infancy

Birth represents a physiological transition rather than a biological reset. The microbial, metabolic, and pharmacological context established during pregnancy extends into the postnatal period through coordinated processes of microbial transmission, nutritional exposure, and maturation of host drug-handling systems. Consequently, infant drug responsiveness reflects not only developmental immaturity but also prenatal programming shaped by microbiota–drug interactions during gestation.

**Figure 3:**
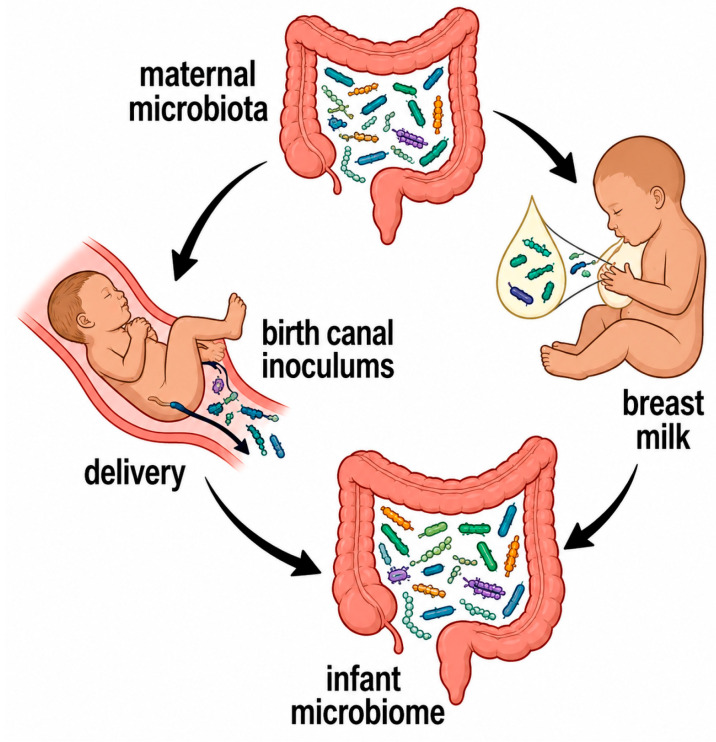
Vertical Transmission (Original schematic created by the authors).

The pharmacological continuity across the maternal–infant axis provides a conceptual framework for bridging the traditional divide between obstetric and pediatric pharmacology. As illustrated in Figure 3, drug exposure during pregnancy may extend beyond maternal effects by reshaping the maternal gut microbiota and its associated metabolic signaling networks, thereby indirectly influencing infant pharmacokinetic and pharmacodynamic profiles. These microbiota-derived genetic and metabolic signals can persist into early life and, through two major routes of vertical microbial transmission delivery and breastfeeding, contribute to the establishment of the infant gut microbiota, ultimately linking maternal drug exposure to infant drug response trajectories.

### 6.1. Vertical Transmission of Microbial and Metabolic Signatures

Initial colonization of the infant gut is strongly influenced by maternal microbial composition at the time of delivery. Mode of birth, peripartum antibiotic exposure, and maternal metabolic status collectively determine the microbial communities that seed the neonatal intestine. Importantly, these communities reflect not only taxonomic composition but also functional metabolic capacity shaped by prenatal exposures [77,78].

Human cohort studies have demonstrated that maternal drug use during pregnancy alters microbial composition and metabolic pathways involved in xenobiotic transformation, short-chain fatty acid production, and bile acid metabolism. Building on these observations, animal model studies further support the inference that infants may inherit microbial ecosystems with modified functional profiles that influence early-life drug handling. However, whether vertical transmission of altered microbial metabolic signatures causally translates into measurable differences in infant drug response remains a hypothesis that requires longitudinal mother–infant pharmacomicrobiomic cohort validation. This proposed mechanism nonetheless provides a biologically plausible link between prenatal pharmacological exposure and postnatal variability in drug response [79,80].

Breast milk further extends maternal microbial and metabolic influence into infancy. Beyond nutrients, breast milk contains microbial metabolites, immune factors, and, in some cases, pharmacological agents or their metabolites. These components interact with the developing infant gut microbiota and immature epithelial and immune systems, reinforcing or modifying prenatal programming. Through this route, maternal microbiota–drug interactions may continue to shape infant physiology long after delivery [81,82,83].

### 6.2. Immature Drug-Handling Systems and Microbiota Dependency in Infancy

Infancy is characterized by incomplete maturation of hepatic drug-metabolizing enzymes, renal clearance mechanisms, and intestinal transport systems. Cytochrome P450 activity, conjugation capacity, and transporter expression evolve rapidly during the first months of life, creating a dynamic and heterogeneous pharmacokinetic landscape [84]. In this context, the gut microbiota assumes a disproportionately influential role in modulating drug bioavailability, efficacy, and toxicity.

In vitro studies using human gut bacterial isolates and in vivo studies in gnotobiotic rodents have demonstrated that microbial metabolism can partially compensate for immature host pathways by transforming drugs or generating metabolites that influence host signaling. Conversely, the same experimental systems have shown that microbial metabolism may produce unexpected bioactive intermediates that alter drug response or toxicity, as exemplified by the well-characterized microbial conversion of levodopa. Extending these findings to the perinatal context, we hypothesize that prenatal programming of microbial composition and function exerts lasting effects on infant pharmacokinetics, contributing to interindividual variability often attributed solely to developmental immaturity. It should be emphasized, however, that this proposition is largely supported by mechanistic and animal evidence; direct longitudinal human evidence in neonatal and infant populations is currently sparse and represents an important research priority [85].

Clinical observations of unpredictable drug responses in neonates and infants, frequently managed through conservative dosing strategies, may reflect unrecognized microbiota-driven modulation. Failure to consider prenatal microbial and pharmacological history risks oversimplifies the determinants of infant drug response and may limit the precision of pediatric pharmacotherapy [86].

### 6.3. Long-Term Implications for Drug Sensitivity and Disease Susceptibility

The convergence of prenatal microbiota–drug interactions, postnatal microbial inheritance, and early-life pharmacological exposure raises the possibility of durable alterations in drug sensitivity across the life course. Experimental models indicate that early perturbations of microbial metabolic signaling can influence immune tolerance, metabolic regulation, and neurodevelopment well into adulthood. Although direct human evidence remains limited, these findings suggest that perinatal pharmacology may shape lifelong pharmacological phenotypes through mechanisms distinct from classical toxicity [87,88].

Importantly, such programming effects are probabilistic rather than deterministic. They reflect altered susceptibility landscapes that may manifest only under specific environmental or pharmacological challenges later in life [89,90]. Recognizing this dimension reframes early-life drug exposure as a contributor to long-term variability in therapeutic response, underscoring the need for longitudinal perspectives in both research and clinical practice.

## 7. Drug Class-Specific Implications Across the Maternal–Infant Continuum

**Figure 4:**
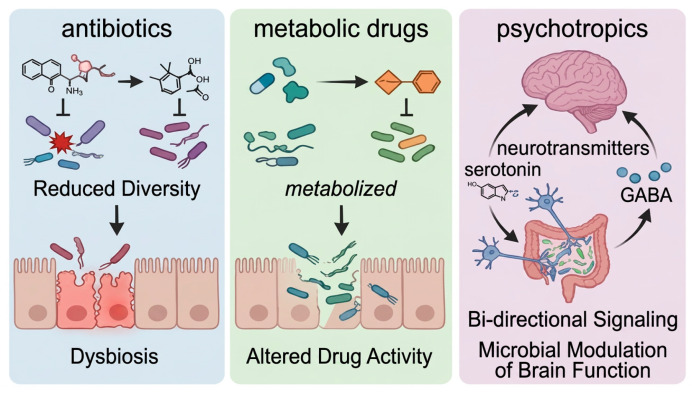
Drug Class Interactions (Original schematic created by the authors).

While microbiota-mediated mechanisms operate across pregnancy and infancy, their impact on pharmacological outcomes is not uniform across therapeutic classes. Differences in molecular targets, metabolic pathways, and capacity to perturb microbial ecology give rise to drug class-specific patterns of developmental programming. Examining representative drug categories illustrates how microbiota-informed principles translate into pharmacological variability across the maternal–infant continuum, as shown in Figure 4 and Table 2.

### 7.1. Antibiotics: Disruption of Microbial Ecology and Developmental Pharmacological Imprinting

Antibiotics represent the most direct and extensively studied perturbation of the maternal gut microbiota during pregnancy. Unlike most therapeutic agents, antibiotics actively reshape microbial community structure and metabolic capacity, often with incomplete recovery over the course of gestation. Prenatal antibiotic exposure has been consistently associated with disproportionate depletion of obligate anaerobic, short-chain fatty acid-producing taxa, including *Bifidobacterium* spp., *Faecalibacterium prausnitzii*, *Roseburia intestinalis*, and members of *Lachnospiraceae* and *Ruminococcaceae* within the Firmicutes phylum, together with reductions in *Bacteroides fragilis* and other *Bacteroidetes* [91,92,93]. Concomitantly, broad-spectrum β-lactams and macrolides frequently produce reciprocal expansion of facultative anaerobes within Proteobacteria, particularly Enterobacteriaceae (e.g., *Escherichia coli*, *Klebsiella* spp.) and *Enterococcus faecalis*, alongside the relative enrichment of antibiotic-resistant *Clostridium* clusters. Aminopenicillins and cephalosporins are most strongly associated with *Bifidobacterium* depletion in neonates, whereas intrapartum prophylaxis with ampicillin or clindamycin disproportionately affects *Bacteroides* and *Lactobacillus* lineages transmitted vertically at delivery. These taxonomic shifts translate into measurable functional consequences, including reduced butyrate and propionate biosynthesis, attenuated BSH activity, and elevated lipopolysaccharide-associated pro-inflammatory tone, which together reshape the pharmacological microenvironment encountered by co-administered drugs.

From a pharmacological perspective, depletion of short-chain fatty acids, producing taxa, alters immune regulation, epithelial barrier integrity, and expression of drug transporters and metabolizing enzymes. These changes may modify the pharmacokinetics of co-administered drugs and influence placental signaling pathways, thereby shaping fetal exposure indirectly. Importantly, antibiotic-induced microbiota disruption may persist beyond drug discontinuation, extending into infancy through altered microbial transmission [94,95]. Postnatally, infants exposed to antibiotics in utero may exhibit heightened sensitivity to pharmacological perturbations due to reduced microbial functional redundancy and immature host drug-handling systems. Antibiotics therefore exemplify how prenatal microbiota disruption can initiate a cascade of pharmacological consequences that extend beyond the immediate therapeutic indication [96].

### 7.2. Metabolic and Endocrine Drugs: Microbiota as a Modulator of Efficacy and Offspring Metabolic Programming

Drugs targeting glucose and lipid metabolism interact with the gut microbiota primarily through bidirectional metabolic and signaling pathways rather than direct antimicrobial activity. Pregnancy-associated insulin resistance and altered lipid handling amplify the regulatory influence of microbiota-derived metabolites, including bile acids and short-chain fatty acids, on host metabolic and pharmacological pathways [97,98,99].

Microbiota-driven modulation of bile acid composition influences nuclear receptor signaling pathways that regulate drug-metabolizing enzymes and transporters. Consequently, the efficacy and clearance of metabolic drugs during pregnancy may vary according to microbial metabolic context and gestational stage. Beyond maternal treatment response, such interactions may influence placental nutrient transport and fetal energy sensing, contributing to long-term metabolic programming in offspring [100].

Animal model studies, primarily in rodents, have consistently shown that prenatal exposure to metabolic drugs alters offspring microbial composition and metabolic phenotype even in the absence of direct postnatal exposure. Corroborating human evidence is emerging from offspring follow-up studies of pregnancies complicated by gestational diabetes treated with metformin or insulin, although these studies are largely observational and confounded by underlying maternal metabolic conditions. Based on this mixed body of evidence, we propose, with appropriate caution given current evidentiary limitations, that microbiota-dependent drug effects during pregnancy may imprint metabolic regulatory circuits with implications for later-life drug responsiveness [101].

### 7.3. Psychotropic and Neuroactive Drugs: Developmental Timing and Microbiota–Gut–Brain Signaling

Psychotropic medications engage the microbiota–gut–brain axis during periods of rapid neurodevelopment, introducing unique considerations for perinatal pharmacology. Several antidepressants and antipsychotics have been shown to alter gut microbial composition and tryptophan metabolism independent of their central nervous system targets. These microbial effects may influence immune signaling, neurotransmitter availability, and placental serotonin pathways [102,103].

Developmental timing is particularly critical for this drug class. Microbiota-mediated modulation of tryptophan–kynurenine pathways during early and mid-gestation may alter fetal neurodevelopmental trajectories without producing immediate toxicity. Postnatally, inherited microbial profiles shaped by prenatal psychotropic exposure may influence stress reactivity and neuroimmune interactions, potentially modifying behavioral responses and drug sensitivity later in life [104,105].

While causal links to long-term neuropsychiatric outcomes remain under investigation, the convergence of neurodevelopmental vulnerability, microbial signaling, and pharmacological exposure underscores the need to consider psychotropic drugs within a microbiota-informed developmental framework [106].

### 7.4. Understudying and Emerging Drug Classes

Many drugs commonly used during pregnancy, including immunomodulators, antiepileptic agents, and biologics, remain poorly characterized from a microbiota-informed perspective. Given their increasing use and potential for long-term developmental impact, systematic evaluation of these classes represents a priority for future research [107,108].

## 8. Methodological Limitations and Critical Knowledge Gaps

Despite rapid advances in microbiome science and developmental pharmacology, the integration of microbiota-informed concepts into perinatal pharmacology remains constrained by substantial methodological and conceptual limitations. Recognizing these challenges is essential for interpreting existing evidence and for guiding future research toward clinically actionable insights rather than associative observations.

### 8.1. Limitations of Current Human Evidence

Most human studies examining drug exposure during pregnancy rely on observational designs, registry-based analyses, or retrospective cohorts. While valuable for identifying population-level associations, these approaches are inherently limited in their ability to resolve mechanistic pathways or disentangle microbiota-mediated effects from genetic, environmental, and socioeconomic confounders. Moreover, microbiota composition is rarely assessed longitudinally across pregnancy, and functional metabolic profiling is often absent [109,110,111].

In many studies, microbial data are collected at a single gestational time point or postnatally, making it difficult to infer temporal relationships between drug exposure, microbial remodeling, and developmental outcomes. Additionally, reliance on taxonomic profiling without parallel metabolomic or transcriptomic analysis obscures the functional relevance of observed microbial shifts. As a result, causal inferences regarding microbiota–drug interactions during pregnancy remain tentative [112].

Ethical and practical constraints further limit direct investigation of fetal exposure and placental mechanisms in humans. Placental samples are typically obtained at delivery, precluding assessment of earlier gestational windows that may be most relevant for developmental programming. These constraints underscore the need for carefully designed integrative studies that combine clinical observation with advanced analytical approaches [113,114].

### 8.2. Challenges in Translational and Experimental Models

Animal models have been instrumental in elucidating mechanistic links between microbiota, drug metabolism, and developmental programming. However, extrapolation to human pregnancy is complicated by species-specific differences in gestational physiology, placental structure, microbial ecology, and drug-handling systems. Rodent models, in particular, differ markedly in placental transport mechanisms and timing of organ maturation, necessitating cautious interpretation [115,116,117].

Germ-free and antibiotic-treated models, while powerful for isolating microbial effects, represent extreme perturbations that may not reflect clinically relevant conditions. The absence of microbial functional redundancy in these models can exaggerate effect sizes and obscure subtler, context-dependent interactions that characterize human pregnancy. Furthermore, standardized drug dosing in experimental systems often fails to capture the variability introduced by gestational stage and microbial metabolic context [118].

Bridging these gaps will require innovative experimental designs that incorporate humanized microbiota, gestational stage-specific interventions, and integrated multi-omics readouts. Such approaches can improve translational relevance while preserving mechanistic resolution [119,120].

### 8.3. Conceptual Gaps in Perinatal Pharmacology

Beyond technical limitations, perinatal pharmacology is constrained by conceptual frameworks that prioritize short-term maternal outcomes and acute fetal toxicity. This focus has historically marginalized subtle, delayed, or probabilistic effects that emerge later in life. Microbiota-mediated developmental programming challenges this paradigm by introducing non-linear, time-dependent mechanisms that operate across biological scales [121,122,123].

Current regulatory and clinical models lack metrics for evaluating long-term pharmacological imprinting or microbiota-dependent modulation of drug response. Without such metrics, clinically meaningful effects may remain invisible to risk–benefit assessments, reinforcing conservative dosing strategies that inadequately address interindividual variability, as shown in Table 3.

## 9. Reframing Perinatal Pharmacology Along the Maternal–Microbiota–Infant Continuum

Perinatal pharmacology has historically been shaped by a reductionist framework that prioritizes short-term maternal safety and immediate fetal outcomes. While this paradigm has successfully mitigated overt teratogenic risk, it is increasingly misaligned with contemporary understanding of developmental biology, systems pharmacology, and host–microbe interactions. The evidence synthesized in this review supports a reconceptualization of pharmacological exposure during pregnancy and early life as a context-dependent, developmentally embedded process rather than a discrete clinical event.

Central to this reframing is recognition of the gut microbiota as an active regulatory component of drug disposition, signaling, and biological impact across gestation and infancy. Maternal microbial communities and their metabolic outputs shape not only maternal pharmacokinetics but also placental function, fetal exposure landscapes, and postnatal drug responsiveness. These effects unfold across interconnected biological interfaces and are highly sensitive to developmental timing. As such, pharmacological perturbations occurring during pregnancy may exert lasting influence even in the absence of detectable short-term toxicity.

This perspective challenges the conventional compartmentalization of obstetric and pediatric pharmacology. Instead, it emphasizes biological continuity across maternal–placental–fetal–infant axis, within which transient perturbations may induce durable programming of metabolic, immune, and pharmacological phenotypes. Translational studies have begun to delineate the maternal–infant microbiota axis as a conduit for metabolic and immune programming, extending prenatal biological signals into postnatal developmental trajectories [124]. Importantly, this programming is not deterministic but probabilistic, shaped by the interaction between drug exposure, microbial metabolic capacity, and host developmental state.

Reframing perinatal pharmacology along this continuum carries significant implications for research and clinical practice. Risk assessment must move beyond static exposure metrics toward developmental trajectories that incorporate gestational timing and microbial context. Mechanistic studies must prioritize functional outputs over taxonomic descriptors, focusing on metabolites, enzyme activity, and signaling pathways that directly interface with host drug-handling systems. Clinically, precision pharmacotherapy during pregnancy and infancy will require integration of microbiota-informed biomarkers with traditional pharmacokinetic and physiological parameters.

Ultimately, advancing perinatal pharmacology will depend on interdisciplinary collaboration that bridges microbiome science, pharmacology, obstetrics, pediatrics, and developmental biology. By embracing a systems-level, developmentally informed framework, the field can move toward therapeutic strategies that achieve immediate clinical goals while safeguarding long-term health trajectories across generations. Integrating microbiota-derived metabolic context into perinatal pharmacology allows drug exposure during pregnancy and early life to be interpreted not as a transient clinical event but as a developmentally programmed determinant of drug response across the life course.

## 10. Conclusions

Perinatal pharmacology is undergoing a conceptual transformation driven by advances in microbiome science and developmental biology. Evidence increasingly indicates that drug exposure during pregnancy and early life cannot be fully understood within frameworks that emphasize transient pharmacokinetic changes and short-term safety endpoints. Instead, pharmacological interventions intersect with dynamic host–microbe ecosystems and developmental processes that shape biological trajectories far beyond the period of exposure.

By integrating microbiota-derived metabolic context into perinatal pharmacology, drug exposure during pregnancy and early life can be understood not as a transient event but as a developmentally programmed determinant of drug response across the life course. This perspective reframes maternal–fetal pharmacology as a continuous, context-dependent process spanning pregnancy and infancy.

Recognizing the microbiota as an active participant in developmental pharmacology opens new avenues for research and clinical innovation. Although substantial challenges remain, incorporating microbial and placental dimensions into pharmacological thinking offers the potential to refine risk assessment, improve therapeutic precision, and ultimately align maternal treatment with long-term offspring health.

## 11. Future Directions

The integration of microbiota-informed principles into perinatal pharmacology necessitates a shift from static exposure-based models toward dynamic, systems-level approaches. Several research and clinical priorities emerge from this perspective.

### 11.1. Longitudinal, Multi-Omics Profiling Across Gestation

Future studies should prioritize longitudinal sampling of maternal microbiota and metabolite profiles across defined gestational stages, coupled with pharmacokinetic and pharmacodynamic measurements. Integration of metagenomics, metabolomics, transcriptomics, and epigenetic profiling can elucidate functional pathways through which microbial metabolism intersects with drug handling and developmental processes. Such datasets will enable identification of microbial metabolic signatures predictive of drug response or developmental outcomes, moving the field beyond descriptive associations toward mechanistic insight.

In addition to metabolite-mediated signaling, the maternal gut microbiota directly transforms xenobiotics through a diverse enzymatic repertoire, including β-glucuronidases, AzoR, nitroreductases, and bile salt hydrolases, which can reactivate hepatically conjugated drugs, drive enterohepatic recirculation, and activate prodrugs such as sulfasalazine and metronidazole. Although gestational stage-specific dynamics of these enzymatic activities remain poorly resolved in human pregnancy, systematic functional characterization across trimesters represents an important direction for future pharmacomicrobiomic research.

### 11.2. Placenta-Centric Experimental and Clinical Models

Given its integrative role, the placenta represents a critical yet underexplored target for microbiota-informed research. Advances in placental organoids, in vivo perfusion systems, and single-cell transcriptomics offer opportunities to investigate how microbial metabolites modulate placental transport, metabolism, and signaling pathways. Incorporating placental endpoints into pharmacological studies can bridge the gap between maternal exposure and fetal outcome, providing a more accurate representation of developmental pharmacology.

### 11.3. Gestational Timing and Context-Specific Dosing Strategies

Recognition of critical windows necessitates gestational stage-specific approaches to drug dosing and monitoring. Rather than applying uniform regimens across pregnancy, future strategies may incorporate microbial metabolic context and developmental timing to optimize efficacy while minimizing long-term risk. Although routine microbiota profiling is not yet clinically feasible, stratification based on readily measurable metabolic or inflammatory markers may offer an interim path toward personalized perinatal pharmacotherapy.

### 11.4. Bridging Obstetric and Pediatric Pharmacology

Microbiota-informed perinatal pharmacology provides a conceptual framework for integrating obstetric and pediatric drug research. Longitudinal cohort studies spanning pregnancy through infancy and childhood will be essential for capturing the continuity of pharmacological programming and its clinical implications. Such integration can inform pediatric dosing strategies and identify early-life predictors of drug responsiveness, improving therapeutic precision across the life course.

## Figures and Tables

**Table 1:** Gestational stage-specific remodeling of microbiota–drug interactions.

Pregnancy	Main Host Physiological Characteristics	Key Changes in the Microbiome	Key Metabolite Changes	Potential Impact on Drug Treatment/Response	Strength of Evidence
Early pregnancy	Immune tolerance establishment, placental formation	Limited diversity changes, primarily functional remodeling	Mild changes in SCFAs and tryptophan metabolites	AhR signaling-mediated CYP enzyme regulation; changes in oral absorption	Mostly animal + limited human
Mid-pregnancy	Insulin resistance, enhanced anabolism	BSH activity alteration; carbohydrate fermentation remodeling	Bile acid spectrum reconstruction (FXR/PXR ligands)	Regulation of CYP3A, UGT, and efflux transporter expression; metabolic drug clearance variants	Human + animal
Late pregnancy	Inflammatory state, blood volume expansion	Microbiome instability; reduction in butyric acid-producing bacteria	Butyrate decreased, LPS exposure increased	Barrier impairment, cytokine-mediated DME/transporter inhibition; placental transport variations	Human observational + mechanistic

**Table 2:** Drug class-specific interactions with the perinatal microbiota.

Drug Class	Microbiota Interaction Mechanism	Biological and Pharmacological Consequences	Implications for Offspring Programming
Antibiotics	Direct disruption of microbial ecology; depletion of obligate anaerobes (e.g., Bifidobacterium, Faecalibacterium); expansion of Proteobacteria.	Reduced SCFA production, attenuated BSH activity, elevated lipopolysaccharide-associated inflammation.	Extended into infancy via altered vertical transmission; shapes early-life drug handling and reduces microbial functional redundancy.
Metabolic and Endocrine Drugs	Bidirectional interaction via bile acids and SCFA signaling pathways.	Microbiota modulates nuclear receptor signaling, modifying the efficacy and clearance of metabolic drugs.	May influence placental nutrient transport and fetal energy sensing, potentially imprinting offspring metabolic regulatory circuits.
Psychotropics and Neuroactive Drugs	Modulation of the microbiota–gut–brain axis and tryptophan–kynurenine pathways independent of CNS targets.	Influences immune signaling, neurotransmitter availability, and placental serotonin pathways.	May alter fetal neurodevelopmental trajectories; postnatally influences stress reactivity and drug sensitivity later in life.

**Table 3:** Limitations of current evidence and key knowledge gaps.

Domain	Current Limitations	Proposed Future Directions
Human Evidence	Heavy reliance on observational/retrospective cohorts; lack of longitudinal assessment; rare use of functional metabolic profiling (often limited to taxonomic data).	Longitudinal, multi-omics profiling across defined gestational stages; integration of metagenomics, metabolomics, and transcriptomics.
Animal and Experimental Models	Species-specific differences in placental structure, organ maturation, and drug handling (e.g., in rodents); germ-free/antibiotic models exaggerate effect sizes.	Utilizing humanized microbiota models; adopting placenta-centric experimental designs.
Conceptual Frameworks	Prioritization of short-term maternal safety and acute fetal toxicity; lack of metrics for long-term pharmacological imprinting.	Reframing risk assessment to include developmental trajectories; bridging obstetric and pediatric pharmacology for a life-course perspective.

## Data Availability

No new data were created or analyzed in this study.
